# Rare causes of exogenous Cushing’s Syndrome: a challenge for endocrinologists

**DOI:** 10.1007/s40618-026-02970-9

**Published:** 2026-06-29

**Authors:** Valentino Marino Picciola, Margherita Medici, Maria Rosaria Ambrosio, Maria Chiara Zatelli

**Affiliations:** 1 Section of Endocrinology, Geriatrics and Internal Medicine, Department of Medical Sciences, University of Ferrara, Via Ariosto 35, Ferrara, 44124 Ferrara Italy; 2 Endocrine Unit, University Hospital S. Anna, Ferrara, 44124 Italy

**Keywords:** Cushing Syndrome, Glucocorticoid, Endocrine disruptor, Supplements, Natural remedies, Review

## Abstract

**Purpose:**

Exogenous Cushing’s syndrome (CS) is most commonly caused by therapeutic glucocorticoids (GC), but rare and atypical sources can mimic endogenous GC. This narrative review provides a concise overview of these uncommon causes and their diagnostic implications.

**Methods:**

A PubMed search was performed using the keywords “Cushing syndrome,” “factitious Cushing syndrome,” “herbal medicine”, “supplements” and “endocrine disruption” on March 9th, 2026. Additional articles were identified through manual screening of reference lists. No restrictions were applied regarding language or study design, and the most relevant publications were selected.

**Results:**

Three main categories of rare exogenous CS were identified. First, herbal or traditional remedies adulterated with undeclared GC. Second, compounds with GC-like activity, which activate the glucocorticoid receptor and suppress the hypothalamic-pituitary-adrenal axis. Third, factitious CS due to covert GC self-administration, often associated with psychiatric or caregiver-fabricated conditions. Diagnosis can be challenging; advanced analytical methods, including liquid chromatography-tandem mass spectrometry (LC-MS/MS) and liquid chromatography-high resolution mass spectrometry (LC-HRMS), are essential to detect synthetic or undeclared GC and differentiate these cases from endogenous hypercortisolism.

**Conclusion:**

Rare exogenous causes of CS represent important diagnostic challenges. Careful evaluation of patient history, combined with the use of LC-MS/MS or LC-HRMS, is crucial to identify these conditions, prevent misdiagnosis, and avoid unnecessary diagnostic or surgical interventions.

## Introduction

**Fig. 1:**
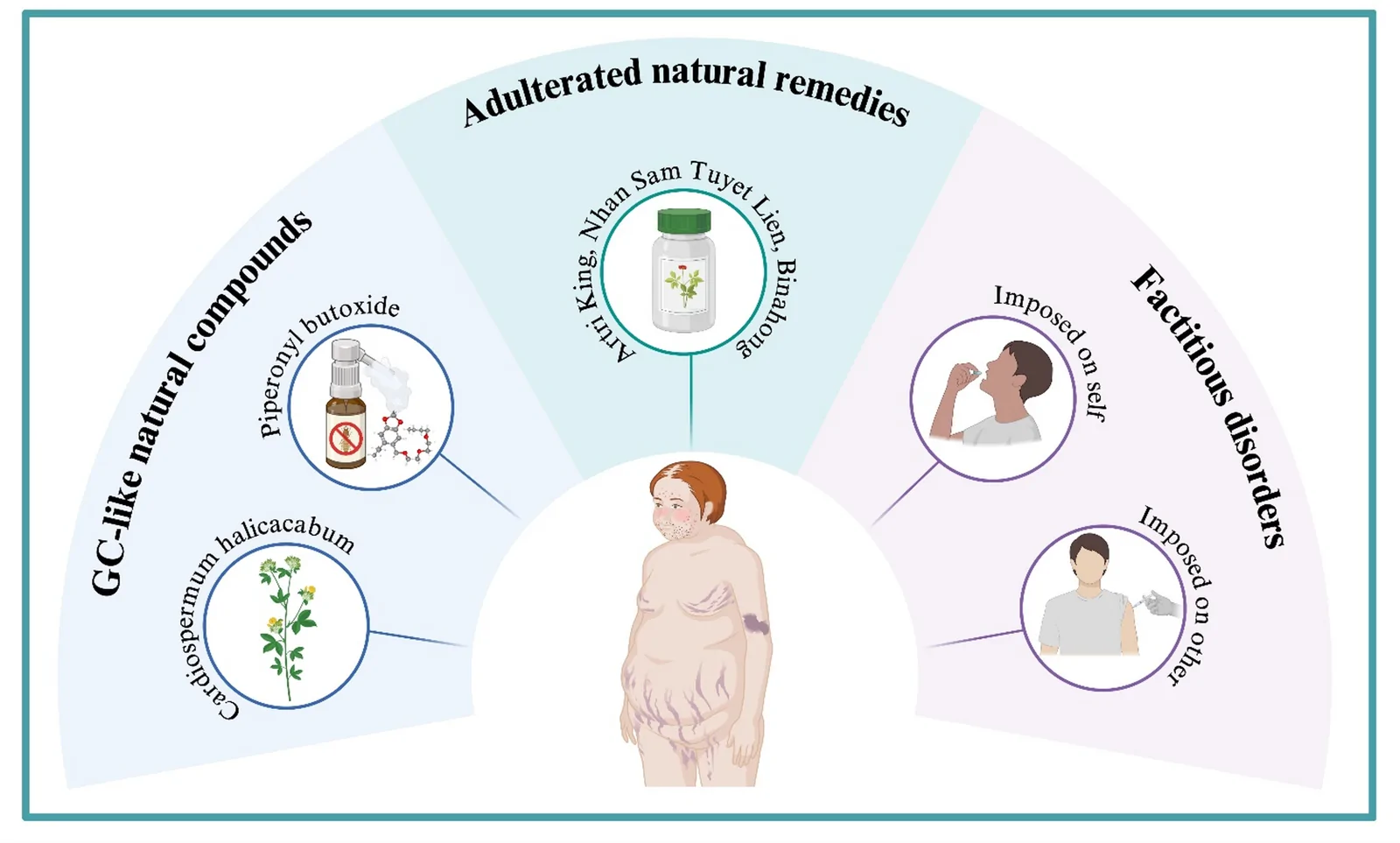
Rare causes of exogenous Cushing’s syndrome

Cushing’s syndrome (CS) is a clinical condition resulting from chronic exposure to excess glucocorticoids (GC). Depending on the source of GC excess, CS can be classified as endogenous or exogenous. The exogenous form represents the most common cause and is typically due to prolonged exposure to supraphysiological GC doses administered for therapeutic purposes, including oral, inhaled, injected, or topical formulations [1–3]. Reflecting their extensive use in clinical practice, approximately 1–3% of the general population worldwide receives these drugs, and chronic use has been reported in 0.5–1.8% of individuals, with prevalence increasing with age [4]. In particular, injectable GCs are commonly used to manage osteoarticular inflammation. Notably, intra-articular or paravertebral triamcinolone acetonide can induce iatrogenic CS and hypothalamic pituitary adrenal axis (HPA) suppression due to its prolonged systemic absorption, a risk amplified by frequent dosing or concurrent CYP3A4 inhibitor drugs [5]. By comparison, endogenous CS is a rare condition caused by autonomous cortisol overproduction, with an estimated incidence of 2–8 cases per million people per year. The main etiologies include ACTH secreting pituitary neuroendocrine tumors, adrenal neoplasms or hyperplasia, and ectopic ACTH or CRH secreting tumors [1–3]. Accordingly, current guidelines recommend excluding exogenous GC exposure as the first step in the diagnostic evaluation of patients with suspected CS [6–8]. Both exogenous and endogenous CS share similar clinical features [3], including plethora, weight gain, rounded face, dorsocervical fat pad, ecchymoses, poor wound healing, striae rubrae, muscle weakness, and hyperandrogenic signs such as hirsutism and acne [1–3]. CS is also linked to significant multisystem complications, including cardiovascular and metabolic dysfunction, increased susceptibility to infections, and neuropsychiatric disorders, all of which substantially impair quality of life [1]. Patients with exogenous CS (ExCS) typically develop clinical signs more rapidly than those with endogenous CS. Although chronic steroid exposure increases the risk of hypertension, these patients often have relatively less severe hypertension and hypokalemia, depending on the mineralocorticoid activity of the administered GC. Moreover, hirsutism and other virilizing features are generally milder than in endogenous CS. ACTH and cortisol production are suppressed in ExCS due to negative feedback on the HPA, which also distinguishes it from endogenous hypercortisolism [3]. Over time, rare and atypical causes of ExCS have been recognized, emphasizing the importance for clinicians to consider the variety of factors contributing to the syndrome in contemporary practice (Fig. 1). This narrative review provides a concise overview of these uncommon causes.

Overview of uncommon sources of exogenous Cushing’s syndrome described in the literature. Created in https://BioRender.com.

## Methods

A literature search was performed in PubMed using combinations of the following keywords: “Cushing syndrome,” “factitious Cushing syndrome,” “herbal medicine,” “supplements” and “endocrine disruption”. On March 9th, 2026 all literature published from database inception was considered. No restrictions on language or study design were applied. Additional articles were identified through manual screening of the reference lists of retrieved publications. Eligible publications included case reports, case series, pharmacovigilance analyses, experimental studies investigating compounds with GC-like activity, and review articles. Publications were selected based on their robustness and completeness of the reported clinical, biochemical, and analytical data. Emphasis was placed on reports providing a clear temporal and causal association between GC exposure and the development of CS features. Preference was given to studies with detailed clinical and laboratory characterization, with higher number of patients, and reports in which the suspected exogenous compound was analytically confirmed using mass spectrometry-based methods. Great importance was assigned to evidence supported by pharmacovigilance data or comprehensive reviews. This review was not conducted as a systematic review.

## Results

**Fig. 2:**
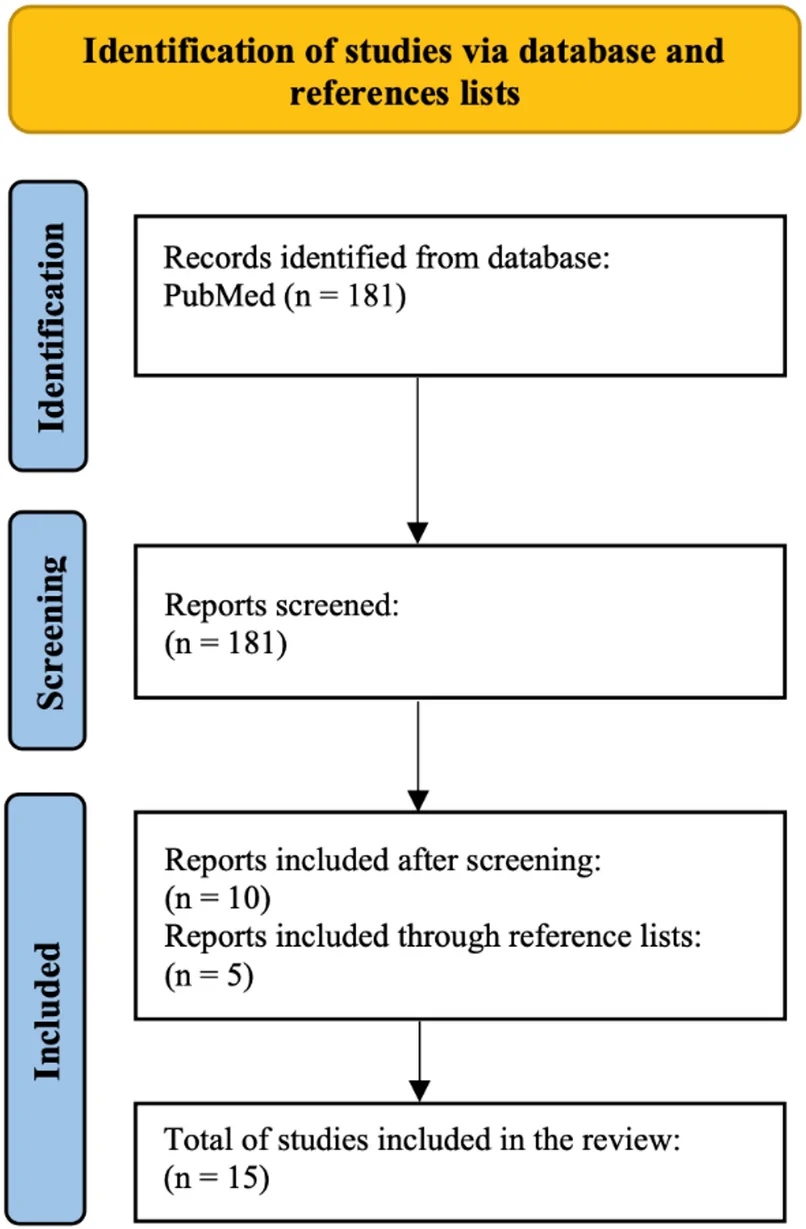
Flowchart of study identification and selection

The literature search initially identified 181 records. After screening titles and abstracts, 15 publications [9–23] were deemed relevant and included in the review. Of these, 5 were identified through manual screening of the reference lists of retrieved articles [9, 13, 15, 17, 19] (Fig. 2). The selected literature was used to identify and describe three main ExCS categories: exposure to adulterated herbal or traditional medicines, non-steroidal compounds with GC-like activity and factitious GC use.

### Adulterated herbal products as a cause of exogenous Cushing’s syndrome

The use of herbal preparations and traditional remedies has increased substantially worldwide over the past decades, involving both developing and high-income countries. This expanding use has raised important concerns regarding safety, quality control, and regulatory oversight of these products. Despite the widespread perception that natural remedies are inherently safe, herbal medicines may be associated with clinically relevant adverse effects. These may result from several factors, including deliberate adulteration with pharmacologically active substances, substitution or misidentification of plant materials, contamination during manufacturing, lack of standardization, inappropriate preparation or dosing, and misleading labeling or advertising practices [9]. Traditional Chinese medicine, which relies predominantly on herbal formulations, represents one of the most widely practiced forms of complementary medicine globally and is commonly used for both disease management and general health promotion, particularly within Chinese communities [10]. Moreover, pharmacovigilance data increasingly highlight the presence of undeclared pharmaceutical compounds in certain proprietary herbal products. In a recent study conducted in Hong Kong, 404 cases of adulterated proprietary Chinese medicines were identified, with GC accounting for 13.8% of the detected undeclared substances. Among patients experiencing adverse reactions, ExCS was reported among the most frequent clinical outcomes [11]. In line with these observations, several case reports have described the occurrence of CS following the use of herbal products or over-the-counter supplements later found to contain hidden GC [12–14]. Commercial preparations such as Artri King, Nhan Sam Tuyet Lien, and Binahong have been implicated in these cases after analytical investigations demonstrated the presence of dexamethasone despite its absence from the declared ingredients [12–14]. The identification of these undeclared compounds has been confirmed by validated analytical techniques, including gas chromatography-mass spectrometry (GC/MS) and liquid chromatography tandem mass spectrometry (LC-MS/MS) [12–15]. These findings underscore the need to consider exposure to undeclared GC contained in adulterated supplements among the potential exogenous causes of CS, particularly in patients reporting the use of herbal or non-prescription health products.

### Non-steroidal compounds with glucocorticoid-like activity

Among uncommon exogenous causes, some cases involve unrecognized exposure to compounds with GC-like activity. In two reported patients presenting with typical clinical CS features, laboratory evaluation excluded endogenous hypercortisolism and demonstrated suppression of the HPA axis consistent with exogenous GC exposure. In both cases, liquid chromatography-high resolution mass spectrometry (LC-HRMS) excluded contamination of the products used with synthetic GC. The first case involved a 15-year-old girl: detailed history revealed chronic use of an over-the-counter anti-lice foam containing natural pyrethrins and piperonyl butoxide (PBO) for approximately one year. The product had been used arbitrarily and beyond the recommended instructions. Serum analysis by LC-HRMS showed markedly elevated PBO concentrations (~ 25-fold higher than in controls) during product exposure. Six months after discontinuation, adrenal function normalized and clinical manifestations resolved. In vitro experiments in HEK293 cells transfected with a GC receptor (GR)-responsive luciferase reporter demonstrated that PBO induced GR-mediated transcriptional activity [16]. A similar mechanism was suggested in a 65-year-old woman who developed CS after chronic daily use for approximately three years of a phytocosmetic cream containing a 10% ethanol extract of *Cardiospermum halicacabum* (CH). Following discontinuation, she developed adrenal insufficiency requiring cortisone acetate replacement, while cushingoid features progressively resolved. In vitro studies showed that CH tincture reduced both basal and CRH-stimulated ACTH secretion in AtT-20/D16v-F2 cells, an effect antagonized by mifepristone. The extract also decreased GR protein expression and directly activated GR-dependent transcription in HEK293 reporter cells, although with slower kinetics and lower potency compared with dexamethasone [17]. These cases illustrate that non-steroidal compounds can exert clinically relevant GC-like effects through GR-mediated mechanisms, sufficient to suppress the HPA axis and induce CS.

### Factitious Cushing’s syndrome

Factitious CS is an exceptionally rare condition caused by the covert GC self-administration with the deliberate intention of producing or simulating clinical features of hypercortisolism. It represents a manifestation of factitious disorder, historically referred to as Munchausen syndrome, and in rare circumstances may occur in the form of caregiver-fabricated illness, in which symptoms are intentionally induced in another individual, typically a child, to obtain medical attention [18, 19]. Only a limited number of cases have been reported in the literature, highlighting the rarity of this entity [12–23]. Available evidence suggests that certain demographic and psychosocial characteristics may be more frequently observed among affected individuals. In a literature review including 23 reported cases of factitious CS, Pineyro et al. found that most patients were women (82%), with a mean age of 37 years. Notably, 43% of the patients had professional or personal connections to the healthcare field, and 57% had a documented history of psychiatric disorders [23]. Diagnostic evaluation may be further complicated by the well-recognized neuropsychiatric manifestations of both exogenous and endogenous CS, which include depression, cognitive impairment, and, in severe cases, psychosis. Consequently, unusual behavior or apparent personality disturbances may be difficult to interpret when distinguishing factitious from endogenous CS [20, 21]. In some reports, patients had prior exposure to the disease through relatives or friends who had undergone evaluation or treatment for CS, potentially facilitating the simulation of clinical manifestations [22]. From a biochemical perspective, case reports and small series indicate that factitious CS often presents with inconsistent laboratory findings. Fluctuating urinary free cortisol levels together with suppressed ACTH concentrations have been described as a common pattern, reflecting intermittent exposure to exogenous GC [22]. The differential diagnosis is particularly challenging because similar biochemical variability may occur in cyclic CS, a rare form of endogenous hypercortisolism characterized by alternating periods of cortisol excess and biochemical remission [18, 24]. In the context of discrepant or unstable laboratory results, highly specific analytical techniques are essential for accurate diagnosis. Conventional immunoassays may exhibit cross-reactivity with synthetic GC or their metabolites, potentially leading to misleadingly elevated cortisol concentrations depending on the specific GC, the timing of exposure, and the residual endogenous cortisol secretion [22]. For this reason, LC-MS/MS is considered the most reliable analytical approach for confirming factitious CS. This technique allows the simultaneous detection of synthetic GC, accurate quantification of endogenous cortisol, and assessment of steroid precursors such as corticosterone, which tend to be increased in endogenous hypercortisolism but not in cases of exogenous GC exposure [18, 21, 22]. Recognition of factitious CS is clinically important not only to establish the correct diagnosis but also to guide appropriate management. Chronic exposure to exogenous GC results in suppression of the HPA axis; therefore, abrupt discontinuation of the offending agent may precipitate secondary adrenal insufficiency. Gradual tapering and temporary GC replacement therapy are often required to ensure safe recovery of adrenal function [18, 21, 24, 25]. Failure to recognize this condition may lead to unnecessary diagnostic procedures or even invasive treatments, including transsphenoidal pituitary surgery or adrenalectomy [21]. Factitious CS therefore represents both a diagnostic challenge and a complex clinical situation that requires careful evaluation and a multidisciplinary approach, including appropriate psychiatric assessment, while maintaining a supportive and nonjudgmental physician-patient relationship [21].

### When to suspect ExCs: a proposal for a diagnostic algorithm

In patients presenting with clinical features suggestive of CS, the initial step consists of a comprehensive and systematic assessment of medication history [1–3, 6–8]. When a clear history of prescribed GC use is identified, the diagnosis of ExCS is generally established, and patient management follows current guideline recommendations [6–8, 25]. In the absence of an overt iatrogenic source, clinicians might want to extend the evaluation to potential covert or unrecognized exposures, including over-the-counter medications, herbal products, dietary supplements, as well as products marketed as “natural” or cosmetic preparations, which may contain undeclared GC or compounds with GC-like activity [9–17]. If such exposure is identified, prompt discontinuation is advisable under clinical supervision, with appropriate monitoring of adrenal function. Temporary GC replacement therapy may be required to prevent secondary adrenal insufficiency due to HPA suppression [9–17, 25]. Analytical techniques such as LC-MS/MS are useful to identify product contamination with synthetic GC and to detect exogenous GC in patients’ samples in suspected cases [9–17]. Conversely, if contamination with known GC is excluded but clinical suspicion of ExCS remains high, clinicians may hypothesize exposure to compounds with GC-like activity [16, 17]. In such cases, the suspected compound may be investigated in biological samples from the patient and compared with appropriate control samples, using targeted analytical approaches [16, 17]. Subsequently, GC-like activity can be assessed through in vitro assays [16, 17]. In cases characterized by fluctuating biochemical results or incongruent clinical and laboratory findings, factitious CS may be considered, particularly in individuals with suggestive psychosocial backgrounds [18–24].

## Conclusions

Although exogenous GC administration for therapeutic purposes remains the most common cause of ExCS, several rare and often overlooked exogenous sources have been increasingly recognized. These include exposure to adulterated herbal or traditional medicines containing undeclared GC, compounds with intrinsic GC-like activity, and factitious GC use. These conditions may closely mimic endogenous hypercortisolism and therefore represent important diagnostic pitfalls. Careful assessment of medication history, including over-the-counter products, supplements, and cosmetic preparations, is essential in patients with suspected CS. Clinicians should also consider all possible administration routes, including oral, inhaled, injected, and topical formulations. In cases with atypical clinical or biochemical findings, advanced analytical techniques such as LC-MS/MS or LC-HRMS can play a crucial role in identifying exogenous compounds or synthetic GC. A greater awareness of these uncommon causes may help prevent misdiagnosis, avoid unnecessary invasive procedures, and ensure appropriate management, including safe withdrawal of the offending agent and monitoring for recovery of HPA function.

## Data Availability

Data sharing is not applicable to this article.
